# Characteristics Associated with Depression Severity in 270 Juveniles in a Major Depressive Episode

**DOI:** 10.3390/brainsci11040440

**Published:** 2021-03-29

**Authors:** Giulia Serra, Maria Elena Iannoni, Monia Trasolini, Gino Maglio, Camilla Frattini, Maria Pia Casini, Ross J. Baldessarini, Stefano Vicari

**Affiliations:** 1 Child Neuropsychiatry Unit, Department of Neuroscience, I.R.C.C.S. Children Hospital Bambino Gesù, 00165 Rome, Italy; mariaelena.iannoni@opbg.net (M.E.I.); monia.trasolini@opbg.net (M.T.); gino.maglio@opbg.net (G.M.); cmllfrt@gmail.com (C.F.); m.casini@policlinicoumberto1.it (M.P.C.); stefano.vicari@opbg.net (S.V.); 2 International Consortium for Mood & Psychotic Disorders Research, Mailman Research Center, McLean Hospital, Belmont, MA 02478, USA; rbaldessarini@mclean.harvard.edu; 3 Department of Clinical and Dynamic Psychology, Medicine and Psychology Faculty, Sapienza University of Rome, 00185 Rome, Italy; 4 Psychiatric Emergency in adolescence Departmental Unit Umberto I General Hospital, 00161 Rome, Italy; 5 Department of Psychiatry, Harvard Medical School, Boston, MA 02478, USA; 6 Child Neuropsychiatry, Catholic University, 00168 Rome, Italy

**Keywords:** adolescent, bipolar, child, depression, mania, mixed, severity, suicidal

## Abstract

**Introduction**: Severe depression is prevalent in young persons and can lead to disability and elevated suicidal risk. **Objectives**: To identify clinical and demographic factors associated with the severity of depression in juveniles diagnosed with a major mood disorder, as a contribution to improving clinical treatment and reducing risk of suicide. **Methods**: We analyzed factors associated with depression severity in 270 juveniles (aged 6–18 years) in a major depressive episode, evaluated and treated at the Bambino Gesù Children’s Hospital of Rome. Depressive symptoms were rated with the revised Children’s Depression Rating Scale (CDRS-R) and manic symptoms with the Kiddie Schedule for Affective Disorders and Schizophrenia (K-SADS) Mania Rating Scale (K-SADS-MRS). Bivariate comparisons were followed by multivariable linear regression modeling. **Results**: Depression severity was greater among females than males (55.0 vs. 47.2), with the diagnosis of a major depressive disorder (MDD) vs. bipolar disorder (BD; 53.8 vs. 49.3), and tended to increase with age (slope = 1.14). Some symptoms typical of mania were associated with greater depression severity, including mood lability, hallucinations, delusions, and irritability, whereas less likely symptoms were hyperactivity, pressured speech, grandiosity, high energy, and distractibility. Factors independently and significantly associated with greater depression severity in multivariable linear regression modeling were: MDD vs. BD diagnosis, female sex, higher anxiety ratings, mood lability, and irritability. **Conclusions**: Severe depression was significantly associated with female sex, the presence of some manic or psychotic symptoms, and with apparent unipolar MDD. Manic/psychotic symptoms should be assessed carefully when evaluating a juvenile depressive episode and considered in treatment planning in an effort to balance risks of antidepressants and the potential value of mood-stabilizing and antimanic agents to decrease the severity of acute episodes and reduce suicidal risk.

## 1. Introduction

Major depressive disorder (MDD) and bipolar disorder (BD) are prevalent psychiatric illnesses that severely limit psychosocial functioning and diminish quality-of-life, with increased risk of fatal outcomes. In 2008, the World Health Organization ranked major depression third as a cause of burden of disease worldwide and projected that it will rank first by 2030 [1]. The Global Burden of Disease Study in 2010 identified depression as the second leading cause of years lived with disability (YLDs), accounting for about 10% of global YLDs and ranking among leading causes of school-absence [2]. BD and MDD also are leading contributors to general health-burden and decreased longevity, associated particularly with suicide and ischemic heart disease [2].

The lifetime prevalence of depression in a community sample of more than 10,000 adolescents aged 12 to 18 years was 11% for major depression, more than a quarter of which (3%) was accounted for by severe depression [3]. The prevalence of depression increased significantly from childhood to late adolescence, especially among females; it peaked at 13% for 15–17 years old females, including 5% with severe depression. Severe depression is associated more with greater risk of psychiatric comorbidity, suicide and functional impairment than are mild or moderate forms [3].

Juvenile mood disorders are highly prevalent, and present an increased risk of suicide, substance abuse, and co-occurring psychiatric and general medical illnesses, with high rates of psychiatric hospitalization and potentially severe and disabling morbidity [4,5,6]. Mood disorders with juvenile onset are reported to be more severe and more recurrent than with adult onset [5,6]. In particular, juvenile onset of MDD has been associated with more later recurrences/year, longer-lasting episodes, and greater symptom-severity, with more agitation or mixed (hypomanic) features, greater suicidal risk, more co-occurring psychiatric and substance-use disorders, as well as lower educational and vocational achievement and marriage rates [6]. Also, several longitudinal studies have found prognosis of juvenile mood disorders to be highly unfavorable, and that their diagnosis and treatment are often delayed for years, especially BD, the onset of which is commonly in adolescent years [7,8]. Such delays probably reflect the difficulty of diagnosing BD at an early age, when clinical presentations often include sub-syndromal depressive or hypomanic symptoms, mood instability, mixed manic–depressive features, a lack of discrete episodes or a very rapidly-cycling course, whereas fully syndromal hypomanic or manic episodes and a clear manic–depressive episodic pattern often arises only later. Also, in a large proportion of cases, the first major mood episode in BD is depressive, making its diagnosis and differentiation from MDD at an early age even more difficult, with potentially risky overuse of antidepressant treatment [9].

Efforts have been made to differentiate bipolar and unipolar depression during childhood and adolescence [10,11] in order to facilitate planning of appropriate, safe and effective treatment. However, accurate differentiation remains challenging in clinical practice, especially when clinicians face the urgent need to quickly reduce affective morbidity as well as to address co-occurring anxiety and substance-use disorders, and suicidal risk.

A critical factor associated with morbidity and disability in juvenile mood disorders is the symptomatic severity of acute episodes. More severe depression has been associated with a generally less favorable prognosis, with an increased risk of hospitalization, co-occurrence of anxiety disorders, poor adherence to treatment, and severe impairment of social, family, and school functioning [12,13], and can lead to economic and legal problems as well as higher suicidal risk [12,13]. Several studies have found that prognosis of juvenile mood disorders is strongly, adversely influenced by the severity of individual acute depressive episodes [1], and that initial depression early in BD is strongly predictive of predominant future depressive morbidity with its associated disability and mortality [14].

Given the apparently raising rates of mood disorders in juveniles [15] and high international rates of suicide associated with major affective disorders in adolescence, we pursued the present study with the aim of identifying factors associated with greater severity of major depressive episodes in children and adolescents diagnosed with either MDD or BD. Such knowledge should help clinicians to identify cases of depression likely to become severe and dangerous, and support the planning of appropriate treatment.

## 2. Methods

### 2.1. Study Subjects

We analyzed factors associated with depression severity in 270 severely ill juveniles diagnosed with a major affective disorder (MDD or BD) at the Mood Disorder Program of Bambino Gesù Children’s Hospital in Rome. The present sample was recruited in a day hospital for the assessment and treatment of early-onset mood disorders. Subjects first were routinely screened in general outpatient clinics and referred to the day hospital if presenting illness was severe enough as to require assessment and treatment in specialized psychiatric program.

Subjects were evaluated at the day hospital during at least 3 appointments, providing a total of 9–10 h of clinical assessment. The evaluation included overall ratings with the Kiddie Schedule for Affective Disorders and Schizophrenia (K-SADS), ratings of symptoms of depression with the Children’s Depression Rating Scale-Revised version (CDRS-R), and ratings of manic symptoms with the K-SADS Mania Rating Scale (MRS). The wording of questions posed could be modified within the guidelines of these questionnaires to improve individual understanding. Evaluations were supplemented with information from family members at more than one visit, as well as by all available documentation of previous evaluations. We also collected information about physiological and pathological history, and education, including psychiatric, medical, social and family history. Current and historical assessments included a retrospective evaluation of antecedent neuropsychiatric symptoms, syndromes, or behavioral abnormalities, with approximate ages at their appearance. All study subjects were assessed by a multi-method procedure that included self-report tools and several semi-structured interviews (anamnestic and diagnostic) with each patient and their family, interviewed both together and separately.

Included subjects were females and males aged 6–18 years diagnosed with a current major depressive episode based on DSM-5 criteria rated with the well-validated Children’s Depression Rating Scale-Revised version (CDRS-R), with a CDRS-R raw score >30 at intake. Excluded were subjects with intellectual disabilities (IQ < 70) or clinical evidence or impoverishment of adaptive or school functioning, or a DSM-5 diagnosis of Autistic Spectrum Disorder.

Parents or legal representatives provided written, informed consent at intake for potential research analysis and anonymous reporting of findings in aggregate form, in accordance with Italian legal and ethical requirements for research uses of clinical data.

### 2.2. Assessments

All study participants were assessed by an experienced child and adolescent psychiatrist and an experienced psychologist, using the Schedule for Affective Disorders and Schizophrenia for School-Age Children-Present and Lifetime Version (K SADS-PL) [16]. This semi-structured, clinician-administered diagnostic interview was performed with both the subjects and their parents or adult legal representatives.

In addition, depressive and manic or mixed symptoms were rated by the same experienced clinicians using the CDRS-R and the K-SADS Mania Rating Scale (MRS), respectively. Also, during assessment visits, the following standard rating scales were scored: Child Depression Inventory (CDI) [17] for self-rating of depressive symptoms; investigator-rated Clinical Global Assessment Scale (CGAS) [18] to evaluate global function; Multidimensional Anxiety Scale for Children (MASC) [19] to assess self-rated anxiety features; Child Behavior Checklist for ages 6–18 (CBCL) [20]. Non-suicidal self-injurious acts, suicidal ideation and suicidal behaviors were evaluated with the Columbia Suicide Severity Rating Scale (C-SSRS) [21].

### 2.3. Structured Psychopathological Assessment

The K-SADS-PL [16] is a semi-structured schedule to assess current and past psychopathological features and psychiatric disorders in juveniles according to the criteria of the Diagnostic and Statistical Manual of Mental Disorders, Fifth Edition (DSM-5) [22]. Study participants and at least one parent or legal guardian were interviewed, to support the collection of all available data via the K-SADS-PL, including substance-abuse related problems and exposure to traumatic experiences.

The Children’s Depression Rating Scale–Revisited (CDRS-R) [23] is a semi-structured interview used to rate depressive symptoms for ages 6–18 years on 17 items (rated 1 to 5 or 7) with raw total scores of 17–113 (given by the sum of the scores at single items and considered positive at scores of >30). The assessment explores 17 scales: school dysfunctions; difficulties in having fun; difficulties in interpersonal relationships; sleep disorders; appetite disorders; excessive fatigue; psychosomatic complaints; irritability; excessive guilt; low self-esteem; depressive feelings; morbid ideas; suicidal ideation; excessive crying; reduced facial expressions; slow speech; and motor hypoactivity.

The Kiddie-SADS Mania Rating Scale (MRS) [24] is a structured interview used to rate manic symptoms for ages 6–18 years on 14 items, with a total score of 1–68 (given by the sum of the scores [1 to 6] for single items minus 13 and is considered positive at total scores of ≥12). Symptoms rated in the MRS are: euphoria and expansiveness, irritability and anger, mood lability, reduced need for sleep, crowded thoughts, increased energy, increased activities, motor hyperactivity, grandiosity, rapid or pressured speech, distractibility, impaired judgment, hallucinations and delusions. MRS total scores of ≥12 are considered clinically significant, with subscale ratings of <2 considered normal, 2–3 borderline, and >3 clinically significant.

The Columbia Suicide Severity Rating Scale (C-SSRS) was used to evaluate suicidal ideation, and has been validated for ages of ≥12 years to assess individual levels of suicidal ideation and behaviors [21].

The Italian version of the Child Behavior Checklist for ages 6–18 years (CBCL-6–18) was completed by each participant and their caregivers to rate behavioral and emotional problems in study subjects. This extensively used tool provides scores with three behavior rating scales that address internalizing symptoms, externalizing symptoms, and total behavioral problems. Sub-items of these three scales include eight syndromal scales (withdrawn–depressed, somatic complaints, anxious–depression, social problems, thought problems, attention problems, rule-breaking behavior, and aggressive behavior). An overall “AAA” CBCL profile was calculated by summing up the scores for attention problems, aggression and anxious–depressed syndromal scales. This score is reported to be indicative of Deficient Emotional Self-Regulation (DESR), at scores of 180–210 (1–2 SD over the mean), and as meeting criteria for a Dysregulation Profile (DP) at a score of >210 (>2 SD above the mean) for the sum of the 3 syndromal scale scores [25].

### 2.4. Statistical Analysis

Averages are reported as means with standard deviation (±SD) or 95% confidence interval (CI). We analyzed factors associated with current depression severity as the CDRS-R raw total score. Preliminary bivariate analyses of factors associated with greater depression severity were based on ANOVA with *t*-scores for continuous variables or contingency tables [χ^2^] for categorical measures. These were followed by stepwise, multivariable, linear regression modeling to provide slope functions (β with CI) and corresponding *t*-scores for association with factors of interest. Analyses were made with commercial statistical software (Stata.13^®^; StataCorp, College Station, TX, USA) using data spreadsheets based on Excel^®^ (Microsoft Corp., Redmond, WA, USA) and Statview.5^®^ (SAS Institute; Cary, NC, USA) software.

## 3. Results

### 3.1. Subject Characteristics

The study sample included a total of 270 juveniles evaluated at the study site in 2017–2019 and diagnosed by DSM-5 as being in an acute major depressive episode. Primary psychiatric diagnoses were major depressive disorder (MDD; *n* = 191), unspecified bipolar disorder (BD-NOS; *n* = 59), type I or II bipolar disorder (BD-I or II, which could not be differentiated reliably; *n* = 20). Family history of any psychiatric illness (66.3%) and mood disorders (56.7%) was identified in a majority of subjects. Current depression evaluated clinically by experienced child and adolescent psychiatrists was considered clinically significant with a CDRS-R score of >30.

The average age at assessment was 14.7 [CI: 14.4–15.6] (range, 6.2–18.0) years; 63% were female (Table 1). The approximate age at onset of first lifetime psychopathological symptoms was 7.55 [CI: 6.96–8.17] (range, 2.00–17.0) years, and the approximate age at onset of first mood symptoms was 12.2 [CI: 11.9–12.5] (range, 2.00–18.0) years (Table 1). The current age ranked by diagnoses are: BD-I/II (15.6 [14.4–16.8]) > MDD (14.8 [14.4–15.2]) > BD-NOS (13.9 [13.0–14.8] years; *t* = 2.03, *p* = 0.018). The onset age, similarly, ranked: BD-I/II (13.4 [12.1–14.7]) > MDD (12.5 [12.1–12.9]) > BD-NOS (10.9 [10.2–11.7] years; *t* = 3.20, *p* < 0.0001). Illness duration (from the onset of mood symptoms to the age at assessment) averaged 2.47 [CI: 2.23–2.71] years (Table 1).

The mean depression severity rating (CDRS-R score) was 52.1 [CI: 50.5–53.7]. Based on an approximate median-split of depression severity ratings, 141 (52.2%) subjects were found to have severe depression (CDRS-R score 62.0 [CI: 60.5–63.5]; range 51–93, Table 1), and 129 (47.8%) had moderate depression (depression score 41.3 [CI: 40.2–42.3]; range 30–50, Table 1).

The average MRS mania rating was 9.98 [CI: 8.89–11.1] (range 1–45). In addition, 66 of the 270 depressed subjects (24.4%) scored as having probable concurrent mania or hypomania, based on an MRS total score of >12, with a corresponding mean total score of 21.1 [CI: 19.9–22.3].

The overall AAA CBCL score averaged 202 [CI: 100–205]; 36.2% of subjects scored >210 (or >2 SD over the mean score), and another 45.7% scored at 180–210 (between 1 and 2 SD over the mean).

All subjects were considered clinically to be severely ill with significant impairment of global functioning as indicated by a mean C-GAS score of 47.3 [CI: 46.4–48.2] (range 20–65). In addition, suicidal ideation in the previous month was reported in 62.7% of the subjects; 41.5% reported at least one lifetime suicide attempt, and 56.4% reported a lifetime history of repetitive non-suicidal self-injurious behavior. A total of 62 subjects (23.2%) had been psychiatrically hospitalized at least once since the onset of their mood disorder.

### 3.2. Factors Associated with Depression Severity

Depression severity was greater among females vs. males (CDRS-R score = 55.0 vs. 47.2; *p* < 0.0001), and somewhat greater in the 191 subjects diagnosed with MDD than in the 79 depressed juveniles diagnosed with BD (53.8 vs. 48.0; *p* = 0.0007). Among BD subjects, depression severity was somewhat greater in BD-I or II than in BD-NOS (52.9 vs. 46.3; *p* = 0.03, Table 2), but did not differ between the 191 subjects diagnosed with MDD and the 20 subjects with BD I or II (53.8 vs. 52.9; *p* = 0.76).

Depression scores were significantly higher with an older current age (regression slope = 1.14 [CI: 0.56–1.73], *p* < 0.0001; Table 2) and with the estimated age at illness-onset (slope = 1.08 [CI: 0.51–1.64], *p* = 0.0002; Table 2).

Depression severity also was greater among subjects with a co-occurring eating disorder (58.2 vs. 51.6; *p* = 0.02), but lower with oppositional–defiant disorder, conduct disorder (52.9 vs. 46.2; *p* = 0.006) or attention (52.5 vs. 42.3; *p* = 0.004) disorder diagnoses (Table 2).

Depression severity also was greater with a history of suicidal ideation within the previous month (depression score = 57.0 vs. 48.9; *p* = 0.001), with a history of repetitive non-suicidal self-injurious behavior (NSSI; 56.9 vs. 49.5; *p* = 0.001), as well as with a lifetime history of at least one probable suicide attempt (57.0 vs. 51.0; *p* = 0.01). In addition, severe depressive episodes were associated with a great likelihood of being psychiatrically hospitalized at least once (depression scores of 59.1 vs. 51.8; *p* = 0.005, Table 2).

Depressive episodes were significantly more severe in association with clinically significant anxiety (based on self-reported MASC anxiety scores; slope, β = 0.29 [CI: 0.16–0.45]; *p* < 0.0001). Depression severity rated by CDRS-R also was greater with a higher self-reported CDI total score of depressive symptoms (β = 0.42 [0.39–0.73]; *p* < 0.0001, Table 2). CDRS-R depression scores also corresponded with scores of several subscales of the CBCL questionnaire, including anxious and depressive measurements, obsessive–compulsive, and post-traumatic problems, and with more deficient emotional regulation (based on self-reported CBCL AAA profile scores (slope β = 0.18 [0.03–0.18]; *p* = 0.005, Table 2).

### 3.3. Manic Features Associated and Depression Severity

Manic symptoms associated with more severe depression were mood lability (CDRS-R depression with feature present vs. absent score= 53.6 vs. 49.2; *p* = 0.008), irritability (CDRS-R score = 53.0 vs. 48.9; *p* = 0.019), as well as psychotic features including hallucinations (CDRS-R score = 58.1 vs. 51.1; *p* = 0.018) and delusions (CDRS-R score = 60.3 vs. 51.4; *p* = 0.031, Table 3). The presence of ≥2 manic symptoms also was associated with greater depression severity (CDRS-R score = 54.1 vs. 49.2; *p* = 0.003, Table 3). Manic symptoms associated with less severe depression included: hyperactivity, pressured speech, grandiosity, increased energy, and distractibility (Table 3). Other manic symptoms were not related to depression severity (Table 3).

### 3.4. Multivariable Linear Regression Model

Factors independently and significantly associated with depression (CDRS-R) scores were: [a] MDD diagnosis (association coefficient, β = 7.83 [3.77–11.9]; *p* < 0.0001); [b] female sex (β = 5.76 [2.12–9.40]; *p* = 0.002), [c] anxiety symptom severity as MASC total score (β = 0.21 [0.07–0.34]; *p* = 0.003); [d] mood lability (β = 4.62 [0.62–8.61]; *p* = 0.024); and [e] irritability (β = 4.44 [0.17–8.71]; *p* = 0.042; Table 4). Standardized coefficients for each variable were: unipolar vs. bipolar (–0.283), females vs. males (0.220), anxiety rating (0.208), lability (0.181), and irritability (0.158). The regression model statistics were the following: F = 11.25; *p* < 0.0001, *r* = 0.509, *r*^2^ = 0.259.

In separate regression modeling of the association of specific MRS items with the diagnosis of MDD vs. BD, the presence of most MRS factors did not differ between the diagnoses. However, increased energy, hyperactivity, and impaired judgment did differ, and all were significantly more prevalent among BD patients (not shown). Additional regression modeling of MRS factors vs. depression severity among BD patients found only rapid thoughts and a tendency for irritability to be associated with more severe depression (not shown). In contrast, MDD subjects had highly significant excesses of delusions and of lability in association with more severe depression, which was not found among BD subjects.

## 4. Discussion

This study analyzed clinical characteristics of 270 juvenile patients for relationships to the severity of their current depression. All subjects had been evaluated initially as outpatients and transferred to a day hospital owing to the severity of their illnesses. Their initial depression ratings by the CDRS-R scale averaged 52.1 [CI: 50.5–53.7], at the 97th percentile for severity. More than half of the sample (141/270 = 52.2%) were considered to have severe depression based on CDRS-R scores of >50, with an average score of 62.0 [60.5–63.6] (Table 1). Study subjects also had high rates of global functional impairment (C-GAS scores <50 in 66.4% [58.0–74.9]), as well as lifetime suicide attempts (41.5% [33.2–49.8]), recent suicidal ideation (within one month, 63.2% [53.8–72.0]), and a history of repeated self-injuries, usually without clear suicidal intent (56.4% [47.5–65.0]) (Table 1). Sui-cidal risks did not differ significantly between subjects diagnosed with MDD or BD, but suicidal ideation, suicide attempts and non-suicidal self-injury were significantly associated with more severe depression ratings across diagnoses (Table 2). Rates of suicidal behavior were higher than in several previous studies of juveniles [26,27,28].

We found several factors to be associated with greater depression severity (CDRS-R scores). Of note, depression was more severe among females, with an older age at onset and current age, and among those that met the diagnostic criteria for MDD rather than a minority (29.3% of cases) of those diagnosed with depressive episodes of BD (Table 2). However, depression severity was similar among patients diagnosed with MDD and BD-I or II, but lower with BD-NOS, to account for the overall lesser severity of depression among BD subjects (Table 1). Greater severity of depression was not associated with any co-occurring psychiatric disorders, with the exception of eating disorders (Table 2). Depression ratings also were more severe among juveniles who had ever been psychiatrically hospitalized (Table 2).

Some previous observations had found more severe depression among juveniles diagnosed with BD compared to MDD cases [10,11]. It is well established that depression is about twice as prevalent among women than men [29,30]. There also are suggestions that depression can be symptomatically different and sometimes more severe in adult women than men [29,30], and that both prevalence and severity of depression may increase with age among juveniles [1,3], as we found (Table 2).

Of particular interest in the present cohort of depressed juveniles were associations of depression severity with clinical features usually associated with mania or hypomania (“[hypo]mania”), as detected with the MRS rating scale. In general, as expected, symptoms of [hypo]mania were more prevalent with BD than MDD, and ranked as: BD-I/II > all BD > BD-NOS > MDD (Table 1). Several MRS items were associated significantly with greater depression severity: multiple MRS items, emotional lability, hallucinations, irritability, and delusions (Table 3). In contrast, other items were associated with lesser severity of current depression: hyperactivity, pressured speech, grandiosity, increased energy, and distractibility (Table 3). Still others were not significantly associated with depression severity: impaired judgment, increased numbers of activities, euphoria, rapid or crowded thoughts, and decreased need for sleep (Table 3). The factors associated with more severe depression can be considered manifestations of psychosis, as might be expected in severe depression, as well as irritability and emotional lability.

Critical questions are whether these associations of more severe depression with [hypo]manic or psychotic features reflect the presence of BD patients in the sample, or possibly indicate the presence of mixed manic–depressive features or states. Based on regression modeling of the association of specific MRS items with diagnoses of MDD vs. BD, most factors did not differ between the diagnostic subgroups. However, increased energy, hyperactivity, and impaired judgment all were significantly and selectively more prevalent among BD patients. Additional regression modeling of MRS factors vs. depression severity among BD patients only found rapid thoughts and a tendency for irritability to be associated with more severe depression. In contrast, MDD subjects had a great excess of delusions and of affective lability in association with more severe depression not found among BD subjects. These observations suggest that the overall association of some MRS factors with more severe depression was not accounted for by the presence of BD subjects. It is also not clear that the presence of psychotic features with more severe depression itself indicates the presence of mixed manic–depressive features; instead it may illustrate the unsurprising association of psychotic features with severe depression.

As a further step to test for the independence of [hypo]manic features with severe depression from the presence of BD, we constructed a multivariable linear regression model that included diagnosis. Factors that remained significantly and independently associated with severe depression were female sex, higher anxiety ratings, affective lability, and irritability, in addition to diagnosis MDD rather than BD. These results add support to the proposal that features usually considered to be characteristic of [hypo]mania were not dependent on the presence of BD patients in the study sample.

An additional consideration of the presence of [hypo]manic features with depression, particularly among subjects considered to have MDD, is that the status of major affective disorders in juveniles continues to have many uncertainties. Indeed, the lack of clear distinction between type I and II BDs in the present subjects, and the presence of a high proportion of unspecified (NOS) BD (74.7% of all BD cases; Table 1) probably reflects uncertainties about the diagnosis of adult-like BD in juveniles, particularly at ages below mid-adolescence, as well as uncertainties about the definition of mixed features or states [31,32]. Subjects diagnosed as BD-NOS were the youngest at intake and the youngest at the estimated age at onset of illness, which is consistent with the view that the syndromal presentation of BD becomes more adult-like with maturation. Moreover, substantial proportions (20–50%) of juveniles presenting with apparent MDD later meet diagnostic criteria for BD, and some with early BD-NOS develop adult-like BD or may have neither BD nor MDD in later years [9,32,33,34]. It is not known whether manic symptoms found with depression in young patients may be predictive of later diagnoses of BD.

Aside from the interpretation of the theoretical significance of some [hypo]manic or psychotic features in association with more severe depression, the presence of such features raises questions about optimally safe and effective treatments of severe depression in children and adolescents. The efficacy of standard antidepressants in juvenile depression is not as secure as in adult major depressive disorder [35,36,37], and the potential role of psychotherapies, alone or combined with pharmacological treatments, for juvenile mood disorders remains underdeveloped [38,39,40,41]. Uncertainties about treatment response combined with uncertainties surrounding the differentiation of MDD and BD in juveniles, and with the present findings of the association of some psychotic or [hypo]manic features in severely depressed children and adolescents, raise the question of the potential value of treatments typical of adult BD for severely depressed young patients. In the treatment of adult depression, either treatment-resistant unipolar depression or bipolar depression, agents usually considered as “mood-stabilizers” (lithium, valproate) or “antipsychotics” (cariprazine, lurasidone, olanzapine + fluoxetine, and quetiapine) have proved useful as monotherapies or in combination with antidepressants [42,43,44,45,46], and in treating BD in juveniles as well as adults [47,48]. Such treatments require further study for the treatment of juvenile depression, particularly in severe cases or those with psychotic or mixed features, who may be at increased risk of adverse outcomes (little benefit and possibly increased suicidal risk) when treated with an antidepressant alone [39,49,50]. An additional therapeutic challenge is the strong association with increased symptoms of anxiety in association with severe depression (Table 2), for which effective treatments remain to be identified.

### Limitations

The present cohort of 270 depressed juveniles consisted mainly of adolescents (*n* = 234, 86.7%) and those diagnosed with MDD (*n* = 191, 70.7%), with only small numbers of prepubertal subjects and those considered to have BD, making balanced comparisons by age and diagnosis difficult. The occurrence of various clinical factors of interest, including co-occurring disorders and the presence of [hypo]manic or psychotic features with depression were based on single, cross-sectional assessments, though they might change over time.

## 5. Conclusions

Severe depression in 270 children and adolescents (191 diagnosed with MDD and 79 with probable BD) was significantly associated with the presence of some symptoms typical of [hypo]mania or psychosis (including delusions, hallucinations, irritability and lability as well as among females, and with apparent unipolar MDD more than with diagnosed BD. Manic and psychotic symptoms should be carefully assessed when evaluating juvenile depressive episodes and be considered in treatment planning, including efforts to balance risks of antidepressants and the potential value of mood-stabilizing and antimanic agents.

## Figures and Tables

**Table 1:** Characteristics of 270 depressed juveniles.

Factor	Measure [95% CI]				
	All Subjects	MDD	All BD	BD-I or II	BD-NOS
Number (n)	270	191	79	20	59
Sex (*n* [%])					
Females	170 [63.0]	134 [70.2]	36 [45.6]	8 [40.0]	28 [47.5]
Males	100 [37.0]	57 [29.8]	43 [54.4]	12 [60.0]	31 [52.5]
Age (years)					
	12.2	12.5	11.6	13.4	10.9
Illness onset	[11.9–12.5]	[12.1–12.9]	[10.9–12.3]	[12.1–14.7]	[10.1–11.7]
	14.7	14.8	14.3	14.3	13.9
Current	[14.4–15.6]	[14.5–15.1]	[13.6–15.0]	[13.6–15.0]	[13.0–14.8]
Illness duration	2.47	2.35	2.77	2.20	2.97
(years)	[2.23–2.71]	[2.06–2.63]	[2.32–3.22]	[1.47–2.93]	[1.82–2.87]
Depression (CDRS-R)					
	52.1	53.8	48.0	52.8	46.3
All (*n* = 270)	[50.5–53.7]	[51.9–55.7]	[45.4–50.6]	[47.2–58.5]	[43.4–49.2]
	62.0	62.8	59.4	60.1	59.0
Severe (*n* = 141)	[60.5–63.5]	[61.1–64.5]	[56.6–62.2]	[54.1–66.1]	[55.7–62.2]
	41.3	41.9	40.2	42.0	39.8
Moderate (*n* = 129)	[40.2–42.3]	[40.5–43.2]	[38.5–41.9]	[37.6–46.4]	[37.9–41.8]
Suicidal risk (%)					
	63.2	65.3	54.6		
Ideation (≤1 month)	[53.8–74.7]	[54.8–74.7]	[32.2–75.6]	–––	–––
	41.5	40.9	44.0	–––	–––
Attempts (lifetime)	[33.2–49.8]	[31.7–50.1]	[24.5–63.5]		
Nonsuicidal	56.4	55.6	60.0	–––	–––
self-injury (%)	[47.5–65.0]	[45.7–65.1]	[38.7–78.9]		
Functional rating	47.3	47.3	47.2	–––	–––
(C-GAS)	[46.0–48.6]	[45.9–48.8]	[44.2–50.2]		
Manic symptoms	9.98	6.65 [6.06–7.24]	18.1	28.4	14.1
(MRS)	[8.89–11.1]		[15.4–20.8]	[22.5–34.3]	[11.9–16.3]
Anxiety symptoms	55.9	56.7 [54.5–58.9]	54.0	56.1	53.4
(MASC)	[54.1–57.7]		[50.8–57.4]	[49.2–63.0]	[49.4–57.4]
AAA profile	202	201	205	205	205
(CBCL)	[199–205]	[198–204]	[199–211]	[193–217]	[198–212]

Depression severity subgroups (severe, moderate) are based on approximate median-split depression severity ratings. Suicidal data are insufficient for bipolar disorder (BD) I vs. II subtypes.

**Table 2:** Factors associated with higher depression ratings in 270 depressed juveniles.

Factor	Depression Severity or Slope [95% CI]	*t*-Score	*p*-Value
Depression Scores [95% CI]			
Sex		4.99	<0.0001
Females	55.0 [53.0–57.0]		
Males	47.2 [45.4–50.6]		
Diagnosis		3.42	0.0007
MDD	53.8 [51.9–55.7]		
BD	48.0 [45.4–50.6]		
Bipolar type		2.22	0.03
BD-I or II	52.9 [47.3–58.5]		
BD-NOS	46.3 [43.4–49.2]		
Comorbidity			
ADHD present	42.3 [38.2–47.1]		
ADHD absent	52.5 [50.9–54.1]	2.92	0.004
ODD present	46.2 [42.8–49.9]		
ODD absent	52.9 [51.3–54.5]	2.76	0.006
ED history	58.2 [53.1–63.7]		
No ED	51.5 [49.9–53.2]	2.33	0.02
Suicidal/Self-harm			
Ideation (≤1 month)	57.0 [54.1–60.1]		
No suicidal Ideation	48.9 [45.0–53.0]		
		3.28	0.001
NSSI present	56.9 [54.1–59.9]		
NSSI absent	49.5 [46.2–53.1]		
		3.29	0.001
Suicide attempt	57.0 [53.9–60.1]	2.61	0.01
No suicide attempt	51.0 [48.0–54.3]		
Hospitalization		2.84	0.005
Present	59.1 [55.0–63.5]		
Absent	51.8 [49.4–54.3]		
Slope [95% CI]			
Age			
Illness onset	1.08 [0.51–1.64]	3.73	0.0002
Current	1.14 [0.56–1.73]	3.84	0.0002
Self-reports			
Depression (CDI)	0.42 [0.39–0.73]	6.53	<0.0001
Anxiety (MASC)	0.29 [0.16–0.45]	4.19	<0.0001
AAA CBCL profile	0.18 [0.03–0.18]	2.82	0.005

Depression severity is rated with the CDRS-R (Children’s Depression Rating Scale-Revised). Abbreviations: AAA CBCL, sum of attention, aggression, and anxiety scores on the Child Behavioral Checklist scale; ADHD, attention deficit-hyperactivity disorder; BD, bipolar disorder; CDI, Children’s Depression Inventory; CI, confidence interval; ED, eating disorder; MASC, Multidimensional Anxiety Scale for Children; NSSI, repeated non-suicidal self-injury; ODD, oppositional-defiant disorder; slope, from bivariate regression vs. depression severity.

**Table 3:** Manic features and depression severity in 270 depressed juveniles.

Mania Factor	Depression Scores [95% CI]		*t*-Score	*p*-Value
	Factor Present	Factor Absent		
**More Depression with Factor Present**				
≥2 Manic features	60.4 [54.6–66.9]	43.9 [37.3–50.1]	3.04	0.003
Lability	53.6 [51.4–55.8]	49.2 [46.8–51.6]	2.67	0.008
Hallucinations	58.1 [52.2–64.0]	51.1 [49.4–52.8]	2.39	0.018
Irritability	53.0 [51.1–54.9]	48.9 [45.9–51.9]	2.36	0.019
Delusions	60.3 [46.9–73.7]	51.4 [49.8–53.0]	2.17	0.031
**Less Depression with Factor Present**				
Hyperactive	46.0 [42.1–49.9]	53.0 [51.2–54.8]	3.30	0.001
Pressured speech	45.0 [41.2–48.8]	52.7 [51.0–54.4]	3.08	0.002
Grandiosity	45.4 [41.0–49.8]	52.4 [50.7–54.1]	2.45	0.015
High energy	46.9 [42.6–51.2]	52.4 [50.7–54.1]	2.20	0.029
Distractibility	46.6 [45.3–51.9]	52.6 [50.7–54.5]	2.02	0.045
**No Difference with Factor Present**				
Impaired judgment	48.7 [45.2–52.2]	52.3 [50.5–54.1]	1.63	0.105
Increased activities	48.7 [43.3–54.1]	52.0 [50.3–53.7]	1.16	0.246
Euphoria	49.2 [44.1–54.3]	52.1 [50.4–53.8]	1.07	0.284
Rapid thoughts	53.2 [49.5–56.9]	51.4 [49.6–53.2]	0.84	0.402
Decreased need for sleep	53.1 [49.7–56.5]	51.5 [49.7–53.3]	0.74	0.460

**Table 4:** Multivariable linear regression model of factors associated with depression severity in 270 depressed juveniles.

Factor	β-Coefficient [95% CI]	*t*-Score	*p*-Value
Unipolar > bipolar	7.83 [3.77–11.9]	3.81	<0.0001
Females > males	5.76 [2.12–9.40]	3.12	0.002
Anxiety rating	0.21 [0.07–0.34]	3.01	0.003
Lability	4.62 [0.62–8.61]	2.28	0.024
Irritability	4.44 [0.17–8.71]	2.05	0.042

Factors not associated with severe current depression included: current age, age at onset, years ill, lifetime history of suicidal ideation or behavior, delusions or hallucinations, any co-occurring psychiatric disorder or their total number, grandiosity, euphoria, distractibility, decreased need for sleep, crowded thoughts, pressured speech, impaired judgment, and hyperactivity. Factors are ranked by significance of association with depression severity. Standardized coefficients for each variable were: unipolar vs. bipolar (–0.283), females vs. males (0.220), anxiety rating (0.208), lability (0.181), and irritability (0.158). The regression model statistics were the following: F = 11.25; *p* < 0.0001, *r* = 0.509, *r*^2^ = 0.259.

## Data Availability

Raw data can be provided for reasonable requests to the first author.
